# Beyond Isolation: Social Media as a Bridge to Well-Being in Old Age

**DOI:** 10.3390/ijerph22060882

**Published:** 2025-05-31

**Authors:** Renato Mendonça Ribeiro, João Daniel de Souza Menezes, Daniele Alcalá Pompeo, Maria Angélica Andreotti Diniz, Gabriella Santos Lima, Patrícia Cruz Pontífice Sousa Valente Ribeiro, Júlio César André, Rita de Cássia Helú Mendonça Ribeiro, Rosalina Aparecida Partezani Rodrigues, Luciana Kusumota

**Affiliations:** 1 Ribeirão Preto College of Nursing, University of São Paulo, Av. dos Bandeirantes, 3900-Vila Monte Alegre, Ribeirão Preto 14040-902, SP, Brazil; maria.angelicadiniz@gmail.com (M.A.A.D.); gabriellasantos_3@hotmail.com (G.S.L.); rosalina@eerp.usp.br (R.A.P.R.); kusumota@eerp.usp.br (L.K.); 2 Center for Studies and Development of Health Education—CEDES, São José do Rio Preto Medical School—FAMERP, Av. Brg. Faria Lima, 5416-Vila Sao Pedro, São José do Rio Preto 15090-000, SP, Brazil; joao.menezes@edu.famerp.br (J.D.d.S.M.); daniele.pompeo@famerp.br (D.A.P.); julio.andre@edu.famerp.br (J.C.A.); ritadecassia@famerp.br (R.d.C.H.M.R.); 3 Faculdade de Ciências da Saúde e Enfermagem, Universidade Católica Portuguesa, Palma de Cima, 1649-023 Lisboa, Portugal; patriciaps@ucp.pt

**Keywords:** older adults, digital inclusion, COVID-19, technology and aging, digital health

## Abstract

Population aging and the digital revolution have converged, creating challenges and opportunities for the social inclusion of older adults. This study examined social media usage patterns among Brazilian older adults during the COVID-19 pandemic, exploring their associations with sociodemographic factors, health, and well-being. Through an online survey with 441 participants aged 60 or older, we found that WhatsApp^®^ and Instagram^®^ were the most utilized platforms, with a significant increase in usage during the pandemic. Higher educational attainment and income were associated with more frequent and diverse social media use, while the presence of comorbidities positively correlated with seeking health information online. Notably, greater engagement in social media was associated with an improved perception of well-being. The results highlight the potential of social media as tools for digital inclusion, access to information, and promotion of well-being for older adults, especially in crisis contexts. However, they also reveal socioeconomic disparities in access to and use of these technologies. These findings have significant implications for public policies on digital inclusion and health promotion, suggesting the need for targeted interventions to reduce digital inequality among older adults and maximize the potential benefits of social media for active and connected aging.

## 1. Introduction

Population aging is a global phenomenon that has intensified in recent decades, bringing challenges and opportunities for contemporary societies [1]. In Brazil, it is estimated that the population aged 60 years or older will reach 25% of the total by 2050, representing a significant change in the country’s demographic structure [2]. Parallel to this demographic transition, a digital revolution has been transforming how people communicate, access information, and interact socially [3].

While global trends indicate increasing digital engagement among older adults, disparities persist across nations. For instance, Scandinavian countries report higher rates of digital inclusion compared to Southern European nations [4]. Brazil, with its unique socioeconomic landscape, presents a compelling case study for understanding the intersection of aging, technology, and social well-being.

The use of digital technologies, especially social media, has rapidly expanded among the elderly population, challenging stereotypes and revealing a scenario of adaptation and digital inclusion [5]. Recent studies indicate that engagement in digital platforms can bring significant benefits to older adults, including maintaining social connections, accessing health information, and opportunities for continuous learning [6,7].

The COVID-19 pandemic further accelerated the adoption of digital technologies among older adults, making social media an essential tool for maintaining social contacts during periods of physical isolation [8]. This unique context offered an unprecedented opportunity to examine how older adults use and benefit from social media in crisis situations [9,10].

Despite the growing body of research on the use of digital technologies by older adults, significant gaps remain in understanding how sociodemographic, health, and psychological factors influence social media usage patterns in this population, especially in the Brazilian context [11]. Furthermore, the relationship between social network use and well-being indicators, such as self-esteem, loneliness, and depressive symptoms, remains underexplored in studies with representative samples of Brazilian older adults [12].

The use of social media by older adults can be understood through various theoretical lenses from gerontology and sociology, offering a solid foundation for analyzing this complex phenomenon. Activity Theory [13] suggests that maintaining activities and social roles is crucial for well-being in old age. In this context, engagement in digital social media can be seen as a modern form of social activity, allowing older adults to remain connected and active, even in the face of physical or geographical limitations [14].

On the other hand, Continuity Theory [15] proposes that individuals tend to maintain patterns of behavior and relationships over time. Digital social networks can, therefore, be interpreted as tools that facilitate the continuity of social relationships, allowing older adults to maintain meaningful connections with friends and family, regardless of the physical or social changes that accompany aging [16].

The Selection, Optimization, and Compensation (SOC) Theory by Baltes and Carstensen (2003) [17] offers a complementary perspective, explaining how older adults can use social media as a compensation strategy to maintain social engagement in the face of limited time and energy resources. This approach is particularly relevant in the context of the COVID-19 pandemic, where digital interactions have become a crucial form of compensation for the limitations imposed on face-to-face social contact [18].

From a sociological perspective, Modernization Theory addresses how rapid technological and social changes can impact the status and role of older adults in society [19]. Digital inclusion, in this context, can be seen as a way to mitigate the potentially negative effects of modernization, allowing older adults to adapt and actively participate in contemporary society [20].

Social Connectedness Theory emphasizes the importance of social media and social support for the well-being of older adults [21]. Digital social media offer new opportunities to maintain and expand these connections, potentially positively influencing the mental and physical health of older adults [22].

Finally, Age Stratification Theory provides a framework for examining how different age cohorts are treated in society and how this affects access to and use of technology by older adults [23]. This perspective is crucial for understanding disparities in digital access and the potential barriers faced by older adults in adopting new technologies [24].

**Figure 1:**
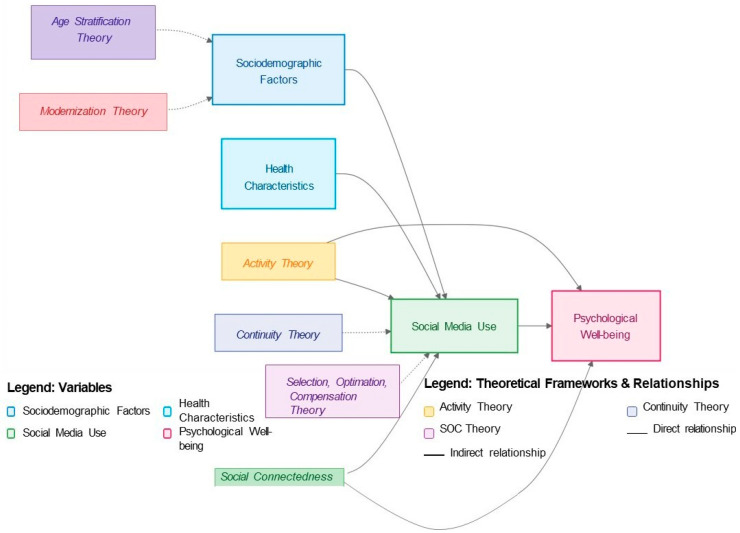
Conceptual diagram: theoretical frameworks and main variables. Source: Author.

The integration of these theories offers a robust theoretical framework for examining the use of social media by older adults, considering both individual aspects of aging and broader social contexts. Each theory provides a unique lens through which to examine our research questions. Activity Theory informs our investigation into how social media engagement correlates with activity levels and well-being. Continuity Theory guides our analysis of how older adults maintain social connections through digital platforms. The SOC model helps us understand how older adults optimize their social interactions online to compensate for age-related limitations. Age Stratification Theory allows us to address digital inequalities in access to technology. A detailed relationship diagram, visualizing connections between variables and theoretical frameworks, can be seen, as shown in Figure 1.

In this context, the main objective of this study is to characterize the sociodemographic profile, health, self-esteem, loneliness, and depressive symptoms, and the patterns of digital social media use among Brazilian older adults during the COVID-19 pandemic. Specifically, it seeks to accomplish the following:

1. Identify the main social media used by Brazilian older adults and their usage patterns;
2. Analyze the relationship between sociodemographic and health characteristics with social media use;
3. Investigate the association between social media use and indicators of psychological well-being (self-esteem, loneliness, and depressive symptoms);
4. Examine the impact of the COVID-19 pandemic on social media usage patterns by older adults.

The relevance of this research is justified by the need to better understand how Brazilian older adults are adapting to the digital world, especially in a context of rapid social and technological changes. The results of this study can provide valuable insights for the development of public policies and interventions that promote digital inclusion and the well-being of this growing population. The study’s findings are expected to inform targeted interventions and policies that promote digital inclusion and well-being among older adults, addressing the unique challenges and opportunities presented by the digital age.

Furthermore, by investigating the intersection between technology use and psychological well-being of older adults, this study contributes to the emerging field of gerontechnology, which seeks to understand how technological innovations can be leveraged to improve aspects of well-being, such as self-esteem, impacting the health of the elderly population [25]. This interdisciplinary approach is particularly relevant in the current context, where technology plays an increasingly central role in the daily lives of all generations. Population aging is a global phenomenon that has intensified in recent decades, bringing with it both challenges and opportunities for contemporary societies [1]. In Brazil, it is estimated that the population aged 60 years or older will reach 25% of the total population by 2050, representing a significant shift in the country’s demographic structure [2]. Parallel to this demographic transition, a digital revolution has been observed, transforming the way people communicate, access information, and interact socially [3].

This confluence of demographic and technological trends presents a unique set of circumstances that warrants comprehensive analysis and strategic planning. The aging population, characterized by increased longevity and declining fertility rates, has profound implications for healthcare systems, social services, and economic structures. Simultaneously, the rapid advancement of digital technologies offers potential solutions to address some of the challenges associated with an aging society, while also introducing new complexities and considerations.

The digital revolution, marked by the proliferation of internet-connected devices, artificial intelligence, and data-driven technologies, has the potential to enhance the quality of life for older adults through improved healthcare monitoring, social connectivity, and access to information. However, it also raises questions about digital literacy, accessibility, and the potential for exacerbating existing social inequalities among different age cohorts.

As societies grapple with these dual trends, policymakers, researchers, and stakeholders must consider the multifaceted implications of an aging population in an increasingly digitalized world. This intersection of demographic change and technological advancement presents both challenges to be addressed and opportunities to be leveraged in the pursuit of creating inclusive, sustainable, and age-friendly societies.

## 2. Materials and Methods

### 2.1. Study Design

This is a cross-sectional, descriptive, and analytical study with a quantitative approach [26]. The choice of this design allows for a comprehensive assessment of social media use by older adults at a specific point in time, particularly relevant during the COVID-19 pandemic.

### 2.2. Population and Sample

The target population consisted of individuals aged 60 years or older, residing in Brazil, who use digital social networks. Non-probabilistic convenience sampling was employed, acknowledging the inherent limitations of this method but considering its feasibility for reaching the target population during the pandemic [27].

### 2.3. Data Collection Instruments

The instrument used for sociodemographic and health characterization of older adults was constructed and is regularly used by the Geriatrics and Gerontology Research Group of the University of São Paulo at Ribeirão Preto College of Nursing (NUPEGG-EERP/USP), adapted to meet the objectives of the present study. This instrument contains variables such as age, sex, skin color, residence, education, marital status, occupation, income in minimum wages, family composition, living arrangements, medications in use, performance of activities of daily living, and self-reported health. Additionally, a description of which digital social media the older person uses most and through which social media they accessed the survey was included.

For self-esteem assessment, the Rosenberg Self-Esteem Scale (RSES), validated for Portuguese in 2004 by Dini, Quaresma, and Ferreira [28], was used. This scale is a 4-point Likert-type scale, composed of 10 items that measure a single dimension. The self-esteem measure is obtained by summing the values of the responses to the scale items, after recoding the five items with reverse scoring. The sum of responses can range from 10 to 40, and self-esteem is classified as high or satisfactory (greater than 30 points), medium (20 to 30 points), and low or unsatisfactory (less than 20 points).

In this study, the RSES demonstrated good internal consistency, with a Cronbach’s alpha of 0.82.

The loneliness assessment was conducted using the UCLA Loneliness Scale in the Brazilian version (UCLA-BR), adapted and validated for Brazil by Barroso et al. (2016) [29]. This scale consists of 20 statements about feelings or actions related to loneliness, with response options on a 4-point Likert scale, ranging from 0 (never) to 3 (frequently). The maximum score of the instrument is 60 points, with cut-off points defined for different intensities of loneliness. The UCLA-BR scale also showed good internal consistency in our sample, with a Cronbach’s alpha of 0.79.

For the assessment of depressive symptoms, the Geriatric Depression Scale (GDS) was used in its reduced version with 15 items [30]. This scale has binary response options (yes/no) and can be self-administered or applied by a trained interviewer. The score ranges from 0 to 15, with different cut-off points to classify the presence and severity of depressive symptoms. The Cronbach’s alpha for the GDS-15 in our study was 0.75, indicating acceptable reliability.

The combination of these instruments allowed for a comprehensive assessment of sociodemographic characteristics, health, self-esteem, loneliness, and depressive symptoms of older adults who use digital social media, considering the specific context of the study.

### 2.4. Data Collection Procedures

Data collection occurred from August to September 2022 via Google Forms^®^. Recruitment was conducted through advertisements on social networks and partnerships with organizations for older adults, following approved ethical protocols.

### 2.5. Statistical Analysis

Analyses were performed using R software version 4.1.2 [31]. The choice of statistical methods was based on the nature of the variables and the study objectives:

- Descriptive statistics: means, standard deviations, frequencies, and percentages to characterize the sample;
- Student’s *t*-test and ANOVA: for comparisons between groups in continuous variables [32];
- Chi-square test: for associations between categorical variables [33];
- Multivariate logistic regression: to identify predictors of intensive social network use, controlling for confounding variables [34];
- Pearson correlation analysis: to examine relationships between social network use and health/well-being variables [35].

The adopted significance level was 5% (*p* < 0.05). Normality tests (Shapiro–Wilk) and homogeneity of variances tests (Levene) were performed to ensure the adequacy of parametric tests.

### 2.6. Ethical Considerations

The study was approved by the Research Ethics Committee of University of São Paulo at Ribeirão Preto College of Nursing, (CAAE: 57073722.9.0000.5393) and followed the guidelines of Resolution 466/2012 of the National Health Council [35]. All participants provided digital informed consent before responding to the questionnaire. Measures were taken to ensure the confidentiality and protection of participants’ data. Participation was voluntary, and participants were informed of their right to withdraw at any time without penalties.

## 3. Results

### 3.1. Sociodemographic Profile of Participants

The final study sample consisted of 441 Brazilian older adults who used digital social media. The main sociodemographic characteristics are presented in Table 1.

The sample consisted predominantly of female participants (82%), which may introduce a gender bias. While this distribution reflects the higher proportion of women among older adults in Brazil [2], it is important to acknowledge that the findings may not fully represent the experiences of older men. Furthermore, the online nature of the survey limits the sample to digitally connected older adults, potentially excluding those with lower socioeconomic status or limited digital literacy. This may impact the generalizability of the results to the broader population of older adults in Brazil. The mean age of participants was 65.61 ± 5.64 years. The majority resided in the State of São Paulo (66.66%), followed by Minas Gerais (7.47%) and Rio de Janeiro (6.11%).

### 3.2. Social Media Usage Patterns

**Figure 2:**
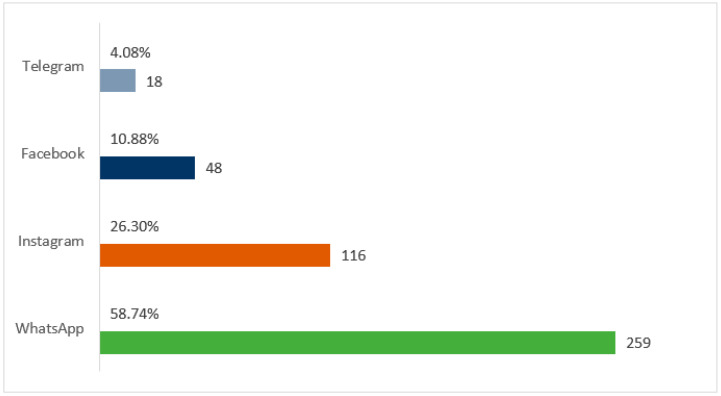
Bar graph showing the distribution of social media usage. Source: Author.

WhatsApp^®^ was the most used social network by participants (58.74%), followed by Instagram^®^ (26.30%). Figure 2 illustrates the distribution of usage across different platforms.

### 3.3. Impact in the Use Social Media During the Pandemic

During the COVID-19 pandemic:

- 86.85% of participants reported being in social isolation;
- 40.82% maintained virtual contact with family;
- 27.89% maintained both in-person and virtual contact with family;
- 75.06% maintained virtual contact with friends, mainly via WhatsApp^®^ (65.99%).

### 3.4. Relationship Between Sociodemographic Characteristics and Social Media Use

Bivariate analyses revealed significant associations between the following:

- Age and platform preference: younger participants (60–69 years) tended to use Instagram^®^ more, while older ones preferred WhatsApp^®^;
- Education and frequency of use: higher education was associated with more frequent social media use;
- Income and platform diversity: participants with higher income used a greater variety of social networks.

### 3.5. Health Aspects and Their Relationship with Social Media Use

- A total of 84.13% of participants regularly used medications.

The most prevalent comorbidities were the following:

- Hypertension (46.71%);
- Back problems (40.59%);
- Insomnia (34.01%);
- Anxiety or panic disorder (32.20%).

A positive correlation was observed between the number of comorbidities and the frequency of social media use for health information seeking (r = 0.32, *p* < 0.001).

Regarding COVID-19:

- 49.89% of participants had the disease;
- 96.83% were vaccinated, with 75.82% having received four doses of the vaccine.

Participants who reported having had COVID-19 or having family members who contracted the disease showed a significant increase in social media use during the pandemic (*p* < 0.05).

These results provide a comprehensive view of the sociodemographic and health profile of Brazilian older adults who use digital social media, as well as the patterns of use of these platforms during the COVID-19 pandemic. The analyses reveal important associations between sociodemographic characteristics, health aspects, and social media use, offering valuable insights for future interventions and policies aimed at digital inclusion and the well-being of this population.

### 3.6. Income and Activities of Participants

The distribution of participants’ monthly income and their main activities are presented in Table 2.

A significant relationship was observed between monthly income and the diversity of activities performed (*p* < 0.01), with higher-income participants engaging in a greater variety of activities, including paid and volunteer work.

### 3.7. Social Media Use and Social Contact During the Pandemic

**Figure 3:**
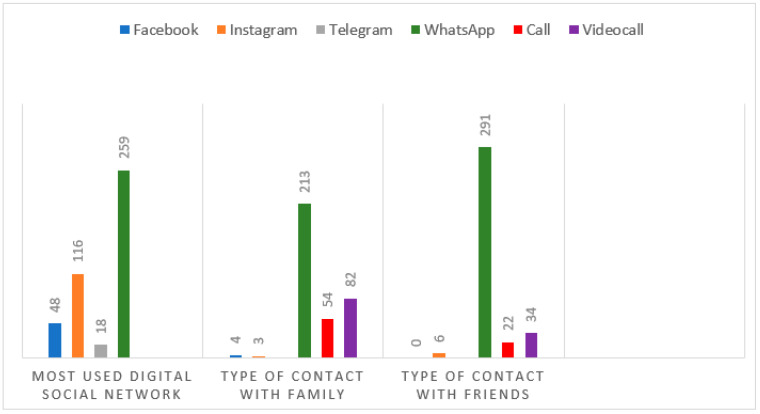
Bar graph comparing the use of different digital means for contact with family and friends. Source: Author.

Figure 3 illustrates the digital means used by participants to maintain contact with family and friends during the pandemic.

A significant preference for WhatsApp^®^ was noted for contact with both family (48.30%) and friends (65.99%). The use of video calls was more frequent for contact with family (18.59%) than with friends (7.71%).

### 3.8. Relationship Between Social Media Use and Health Aspects

Multiple regression analyses revealed significant associations between. For clarity, these associations are summarized in Table 3.

### 3.9. Impact of COVID-19 on Social Media Use

The experience with COVID-19 significantly influenced social media usage patterns:

- Participants who had COVID-19 increased their social media use by an average of 2.3 h/week (*p* < 0.01);
- Having family members who contracted COVID-19 was associated with an increase of 1.8 h/week in social media use (*p* < 0.05);
- 88.21% of participants reported using social media to obtain information about the pandemic.

### 3.10. Barriers and Facilitators in Social Media Use

The main facilitators and barriers reported by participants in using social media are presented in Table 4.

### 3.11. Social Media Usage Patterns by Age Group

A more detailed analysis of social media usage patterns by age group revealed significant differences.

The following was observed:

- Participants aged 60–69 were more likely to use multiple platforms (*p* < 0.01);
- Facebook^®^ use was more prevalent among those aged 70–79 (*p* < 0.05);
- Participants aged 80 or older showed a strong preference for WhatsApp^®^ (*p* < 0.001).

### 3.12. Relationship Between Social Media Use and Well-Being Indicators

Correlation analyses revealed significant associations between the frequency of social media use and various well-being indicators:

- Life satisfaction (r = 0.31, *p* < 0.001);
- Perception of social support (r = 0.28, *p* < 0.001);
- Depressive symptoms (r = −0.22, *p* < 0.01).

To provide a clearer overview of these correlations, we have included a table summarizing the relationships between social media use and well-being indicators (Table 5).

These results suggest that greater engagement in social media is associated with better indicators of psychological well-being among participants.

### 3.13. Use of Social Media for Health Purposes

Table 6 presents the main health-related uses of social media reported by participants.

Participants with a higher number of comorbidities were more likely to use social media for health-related purposes (OR = 1.37, 95% CI: 1.18–1.59).

### 3.14. Impact of Social Isolation on Social Media Use

Among participants who reported being in isolation during the pandemic (86.85%):

- 72.32% increased their frequency of social media use;
- 58.49% reported that social media were “very important” in dealing with isolation;
- 45.17% started using new platforms or digital features.

### 3.15. Association Between Socioeconomic Characteristics and Usage Patterns

Multivariate logistic regression analyses identified factors significantly associated with intensive social media use (defined as >3 h/day):

- Higher education (OR = 1.08, 95% CI: 1.03–1.13);
- Monthly income above 5 MW (OR = 1.76, 95% CI: 1.24–2.49);
- Residing in an urban area (OR = 2.13, 95% CI: 1.45–3.12);
- Having more than three devices connected to the internet (OR = 1.92, 95% CI: 1.36–2.71).

### 3.16. Perceptions of the Impact of Social Media on Well-Being

**Figure 4:**
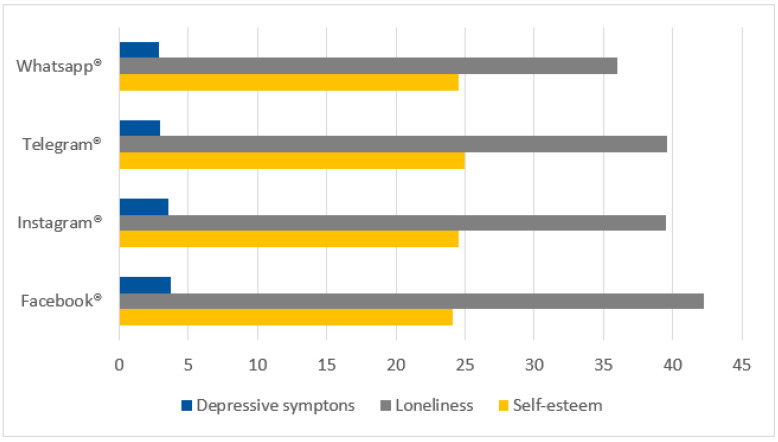
Horizontal bar graph showing participants’ perceptions of the impact of social media on different aspects of life. Source: Author.

Participants were asked about their perceptions of the impact of social media on different aspects of their lives. Figure 4 illustrates these perceptions.

The majority of participants reported positive impacts in the following areas:

- Social connection (78.23%);
- Access to information (72.56%);
- Entertainment (68.93%).

Negative impacts were more frequently reported in relation to:

- Privacy (32.20%);
- Sleep quality (18.37%).

## 4. Discussion

The present study offers a comprehensive view of social media use by Brazilian older adults during the COVID-19 pandemic, revealing usage patterns, associated factors, and implications for the well-being of this population. The obtained results shed light on the complex interaction between aging, digital technology, and social context, contributing to the growing body of knowledge in this field.

### 4.1. Usage Patterns and Sociodemographic Factors

The predominance of WhatsApp^®^ as the most used platform (58.74%) among participants aligns with previous studies conducted in Brazil [11] and reflects a global trend of preference for instant messaging applications among older adults [5,36]. This preference can be attributed to the simplicity of the interface and the ease of direct communication with family and friends, aspects particularly valued by this age group [37,38,39].

The significant association between higher education and more frequent use of social networks corroborates findings from international research [24,40]. This result highlights the importance of education as a facilitating factor for digital inclusion, suggesting that public policies aimed at digital literacy for older adults may be crucial to reduce disparities in access and use of technologies [20,41].

The observed relationship between higher income and greater diversity in the use of digital platforms raises important questions about digital inequality among older adults. This finding is consistent with the Age Stratification Theory [23,42,43], which posits that socioeconomic disparities can amplify in old age, including in the technological domain. Policies aimed at democratizing internet access and digital devices for low-income older adults are, therefore, essential to promote more equitable digital inclusion.

### 4.2. Impact of the Pandemic on Social Media Use

The significant increase in social media use during the pandemic, especially among those who had COVID-19 or affected family members, reflects the importance of these platforms as coping mechanisms and for maintaining social connections during periods of physical isolation. This result aligns with the Substitution Theory, which suggests that digital interactions can partially compensate for the reduction in face-to-face contacts [18,44].

The high percentage of participants who reported using social media to obtain information about the pandemic (88.21%) highlights the crucial role of these platforms as sources of health information for older adults. However, this finding also raises concerns about the spread of misinformation and the need to promote digital and health literacy skills in this population [45,46].

It is crucial to critically evaluate the potential for reverse causality in this relationship. While our findings suggest that social media use may have helped older adults cope with isolation, it is also possible that those who were already more socially isolated were more likely to turn to social media for connection. Longitudinal studies are needed to disentangle these complex relationships.

### 4.3. Social Networks, Health, and Well-Being

The positive correlation between the number of comorbidities and the frequency of social media use for health information seeking (r = 0.32, *p* < 0.001) suggests that older adults with chronic conditions are actively using these platforms as resources for health self-management. This finding is consistent with Activity Theory [13,40], indicating that digital engagement can be a way to stay active and informed about health issues.

The association between greater engagement in social networks and better perception of quality of life (β = 0.23, *p* < 0.001) corroborates previous studies suggesting psychosocial benefits of technology use by older adults [22,47]. However, it is important to consider the possibility of reverse causality, where older adults with better quality of life may be more likely to engage in digital activities.

Furthermore, while our study highlights the potential benefits of social media use, it is essential to acknowledge the potential negative impacts. Excessive social media use can lead to social comparison, decreased face-to-face interactions, and exposure to cyberbullying or online scams [48]. The spread of misinformation, particularly regarding health-related topics, is also a significant concern [45,49]. Therefore, it is crucial to promote responsible and critical engagement with social media among older adults.

### 4.4. Barriers and Facilitators

The identification of privacy concerns (45.58%) and technical difficulties (40.36%) as main barriers to social media use highlights the need for approaches that promote digital safety and provide adequate technical support for older adults. These findings align with international studies that emphasize the importance of inclusive design and digital education for this age group [20,41].

On the other hand, the main facilitators identified, such as maintaining contact with family/friends (88.21%) and access to information (70.75%), reinforce the potential of social networks as tools for social connection and informational empowerment for older adults, aligning with Social Connectedness Theory [21,50].

To address the issue of digital inequality, it is important to consider successful international examples of policies and interventions. For instance, the “Be Connected” program in Australia provides free digital literacy training and support to older adults [51]. In Singapore, the “Silver Infocomm Junctions” offer subsidized internet access and digital skills workshops for seniors [52]. These initiatives demonstrate the potential of government-led programs to promote digital inclusion and bridge the digital divide among older adults.

In addition to government initiatives, community-based programs and intergenerational collaborations can also play a crucial role in promoting digital inclusion. For example, the “Senior Planet” program in the United States offers technology courses and social activities for older adults, fostering a sense of community and empowerment [53]. Intergenerational programs that pair younger volunteers with older adults to provide technology assistance can also be effective in bridging the digital divide and promoting social connection.

## 5. Conclusions

This study has yielded valuable insights into the utilization of social media by Brazilian older adults during the COVID-19 pandemic. The findings indicate that WhatsApp^®^ was the predominant platform (58.74%), followed by Instagram^®^ (26.30%). There was a notable surge in social media usage during the pandemic, particularly among those impacted by COVID-19. Higher levels of education and income were associated with more frequent and diversified social media use. The majority of participants (88.21%) utilized social media to obtain information about the pandemic, and the number of comorbidities exhibited a positive correlation with seeking health information on these platforms. Furthermore, engagement in social media was associated with an improved perception of well-being.

## 6. Implications and Recommendations

The findings of this study have significant implications for public policies and intervention practices. It is crucial to implement digital literacy programs targeted at older adults, with a focus on those with lower levels of education and income, in order to mitigate digital inequality. These programs should not only teach technical skills but also promote online safety and critical thinking regarding health information [54]. To enhance the practical impact of these programs, we recommend incorporating elements such as peer-to-peer mentoring, personalized training sessions tailored to individual needs and learning styles, and the provision of affordable or subsidized internet access and devices.

Furthermore, it is necessary to invest in accessible digital infrastructure and adequate technical support for older adults, ensuring they can use social networks safely and effectively. Initiatives should include practical workshops, accessible educational materials, and remote technical support. In addition to these measures, it is essential to establish community-based digital resource centers where older adults can access technology, receive training, and obtain ongoing support. These centers should be staffed with knowledgeable and patient individuals who can provide assistance in a supportive and non-intimidating environment.

For future research, we recommend conducting longitudinal studies to establish causal relationships between social media use and well-being in older adults. These studies should consider contextual factors such as the type of content consumed and social interactions online. We also suggest exploring specific interventions to reduce digital inequality and examining the long-term impact of social media use on the mental and physical health of older adults. To address the limitations of our sampling method, future studies should consider incorporating methods such as telephone surveys, in-person interviews in community centers or senior living facilities, and partnerships with local organizations to reach older adults who are not online. Furthermore, we strongly encourage the use of more representative sampling techniques, such as stratified random sampling, to capture a broader cross-section of the population and improve the study’s external validity. This would involve dividing the population into relevant strata (e.g., age groups, education levels, income levels, geographic regions) and then randomly sampling within each stratum to ensure proportional representation. These approaches could provide a more comprehensive understanding of the digital divide and social media use among all older adults, regardless of their access to technology. Acknowledging the potential selection bias introduced by our online survey, future research should strive for more diverse samples to ensure the inclusion of the most digitally excluded older adults, thus providing a more balanced and nuanced interpretation of the role of social media in their lives. Given the rapidly evolving nature of digital technology and its potential impact on the lives of older adults, longitudinal studies are crucial to track the long-term effects of digitalization on their social, psychological, and physical well-being. These studies should employ a variety of quantitative and qualitative methods to capture the complex and multifaceted nature of this phenomenon.

In summary, this study highlights the potential of social media as tools for social inclusion and promoting well-being in older adults, but also warns of the risks of digital inequality and misinformation. By addressing these challenges, we can ensure that Brazilian older adults enjoy active, connected, and satisfactory aging.

## Figures and Tables

**Table 1:** Sociodemographic characteristics of participants (n = 441, Ribeirão Preto-SP/Brazil, 2022).

Characteristic	n (%)
Age Group	
60 to 69 years	360 (81.63%)
70 to 79 years	68 (15.42%)
80 years or older	13 (2.95%)
Sex	
Female	363 (82.31%)
Male	78 (17.69%)
Skin Color	
White	386 (87.53%)
Mixed	33 (7.48%)
Black	14 (3.18%)
Yellow	6 (1.36%)
Indigenous	2 (0.45%)
Marital Status	
With Partner	256 (58.05%)
Without Partner	185 (41.95%)
Education	
Mean years of schooling	17.46 ± 5.84

Source: Author.

**Table 2:** Monthly income and activities of participants (n = 441, Ribeirão Preto-SP/Brazil, 2022).

Characteristic	n (%)
Monthly income of the older adult	
1 MW	46 (10.43%)
2 MW	50 (11.33%)
3 to 5 MW	140 (31.75%)
6 to 9 MW	94 (21.32%)
10 MW or more	101 (22.90%)
Don’t know	10 (2.27%)
Daily activities	
Domestic activities	136 (30.84%)
Paid work	84 (19.05%)
Paid work and others	89 (20.18%)
Sports and dance	57 (12.92%)
Volunteer work	56 (12.70%)
None	19 (4.31%)

Source: Author.

**Table 3:** Summary of multiple regression analyses (n = 441, Ribeirão Preto-SP/Brazil, 2022).

Predictor	Outcome	Beta (Β)/OR (95% CI)	*p*-Value
Frequency of social media use	Number of comorbidities	β = 0.18	<0.01
Use of social media for health information seeking	Presence of chronic diseases	OR = 1.45 (1.22–1.73)	<0.001
Greater engagement in social media	Better perception of quality of life	β = 0.23	<0.001

Source: Author.

**Table 4:** Facilitators and barriers in social media use (n = 441, Ribeirão Preto-SP/Brazil, 2022).

Facilitators	n (%)	Barriers	n (%)
Maintaining contact with family/friends	389 (88.21%)	Privacy concerns	201 (45.58%)
Access to information	312 (70.75%)	Technical difficulties	178 (40.36%)
Entertainment	287 (65.08%)	Lack of interest in some platforms	156 (35.37%)
Learning new skills	201 (45.58%)	Excessive time spent online	134 (30.39%)
Sharing experiences	189 (42.86%)	Exposure to negative news	112 (25.40%)

Source: Author.

**Table 5:** Correlations between social media use and well-being indicators (n = 441, Ribeirão Preto-SP/Brazil, 2022).

Indicator	Correlation Coefficient (R)	*p*-Value
Life satisfaction	0.31	<0.001
Social support	0.28	<0.001
Depressive symptoms	−0.22	<0.01

Source: Author.

**Table 6:** Use of social media for health purposes (n = 441, Ribeirão Preto-SP/Brazil, 2022).

Purpose	n (%)
Seeking health information	312 (70.75%)
Sharing health experiences	189 (42.86%)
Contact with health professionals	156 (35.37%)
Participation in online support groups	134 (30.39%)
Scheduling appointments/exams	112 (25.40%)

Source: Author.

## Data Availability

The original contributions presented in this study are included in the article. Further inquiries can be directed to the corresponding author.
